# High Red Meat Intake Exacerbates Dextran Sulfate-Induced Colitis by Altering Gut Microbiota in Mice

**DOI:** 10.3389/fnut.2021.646819

**Published:** 2021-07-20

**Authors:** Dan-ping Li, Min Cui, Fang Tan, Xiao-yan Liu, Ping Yao

**Affiliations:** ^1^Department of Gastroenterology, The First Affiliated Hospital of Xinjiang Medical University, Ürümqi, China; ^2^Department of Gastroenterology, The Fifth Affiliated Hospital of Xinjiang Medical University, Ürümqi, China

**Keywords:** gut microbiota, red meat diet, inflammatory bowel disease, dextran sulfate, ulcerative colitis

## Abstract

Inflammatory bowel disease (IBD) is a serious hazard to public health, but the precise etiology of the disease is unclear. High intake of red meat diet is closely related to the occurrence of IBD. In this study, we investigated whether the high intake of red meat can increase the sensitivity of colitis and the underlying mechanism. Mice were fed with different levels of red meat for 8 weeks and then the colonic contents were analyzed by 16S rRNA sequencing. Then 3% dextran sulfate sodium was used to induce colitis in mice. We observed the severity of colitis and inflammatory cytokines. We found that high-dose red meat caused intestinal microbiota disorder, reduced the relative abundance of *Lachnospiraceae_NK4A136_group, Faecalibaculum, Blautia* and *Dubosiella*, and increased the relative abundance of *Bacteroides* and *Alistipes*. This in turn leads to an increase in colitis and inflammatory cytokine secretion. Moreover, we found that high red meat intake impaired the colon barrier integrity and decreased the expression of ZO-1, claudin, and occludin. We also found high red meat intake induced the production of more inflammatory cytokines such as IL-1β, TNF-α, IL-17, and IL-6 and inflammatory inducible enzymes such as COX-2 and iNOS in dextran sulfate sodium-induced colitis. These results suggest that we should optimize the diet and reduce the intake of red meat to prevent the occurrence of IBD.

## Introduction

The gut microbiota is an indispensable part of the human body. It evolves with the host and acts as a natural barrier to help the body digest food, absorb nutrients and maintain health (1). After cessation of breastfeeding during childhood, the gut microbiota begins to stabilize and become a stable system. Studies have clarified the importance of relationships between microbiota and host in early life, and these interactions have been further confirmed in later specific disease states (2), such as diabetes, asthma (3) and colitis (4). Intestinal microbiota imbalance can affect host metabolism, immunity and health, and induce many diseases such as inflammatory bowel disease (IBD), colorectal cancer (5), obesity (6), type 1 and type 2 diabetes (6), and weakened immunity among the elderly.

Crohn's disease and ulcerative colitis are the most common subtypes of inflammatory bowel disease and can be differentiated based on their clinical presentation (including disease location and symptoms) as well as their histopathology. The incidence of IBD is increasing worldwide, but the exact cause of the disease remains unclear. Studies have confirmed that excessive intake of red meat is associated with an increased incidence of IBD (7–9). A cohort study of 67,511 people in France showed that high-protein, high-fat diets of red meat were significantly associated with the onset of IBD (8). Another nutritional study suggests that high meat intake, especially red meat, increases the risk of UC and CD (9). In a prospective study on cancer and nutrition in Europe, red meat, margarine, sunflower oil and corn oil were found to contain large amounts of omega-6 unsaturated fatty acids, while omega-6 unsaturated fatty acids have been shown to increase UC risk (10). In another prospective cohort study, red meat and processed meat were associated with a risk of UC recurrence (11). We also found the incidence of IBD was higher than other places in Xinjiang of China, which was believed to be related with their custom of daily high red meat intake for a long time.

Red meat mainly contains protein, fat and heme. In general, high protein intake causes disturbances in the gut microbiota, which in turn leads to changes in metabolites such as branched-chain fatty acids, amino acids and hydrogen sulfide. These all affect the viability and proliferation of epithelial cells, intestinal barrier function and immune response. In addition, high fat intake also changes the intestinal microbiota, thereby increasing intestinal mucosal permeability, reducing the expression of tight junction proteins in the intestinal epithelium, and exacerbating intestinal inflammation. Heme can mediate inflammatory stress responses in Gram-negative bacteria (12). Constante M et al. reported dietary heme can affect the gut microbial community structure and contribute to microbiota dysbiosis (13). Therefore, we hypothesize that high intake of red meat can cause damage to the gut microbiota, which in turn exacerbates inflammation.

In this study, we assessed the effects of red meat diet on gut microbiota composition in mice and their subsequent sensitivity to DSS (dextran sulfate sodium)-induced colitis.

## Materials and Methods

### Animals

Three-week-old male and female BALB/c mice were purchased from Beijing Vital River Laboratory (Beijing, China). All animals were housed in specific pathogen-free conditions and in a 12-h light/dark cycle. All animal experiments complied with the ethical guidelines of People's Republic of China. The experimental protocol was approved by the Ethics Committee of Xinjiang Medical University (approval no. IACUC20180411-04).

### Diets and Experimental Design

Red meat diet was made from beef powder (Weifang meibaolai animal protein Co., Ltd. China), containing about 70–78% protein, 10–12% fat, 10% water and others. All diets were prepared synthetically from Jiangsu Xietong Pharmaceutical Bio-engineering Co., Ltd (Jiangsu, China). The ingredients of the experimental diets were shown on Table 1.

**Table 1:** Composition of different levels of red meat diets.

**Ingredient**	**Control**	**LD**	**MD**	**HD**
Casein (g/kg)	200	0	0	0
Beef (g/kg)	0	250	500	750
Corn starch (g/kg)	397.486	347.486	97.486	0.486
Sucrose (g/kg)	100	100	100	100
Maltodextrin (g/kg)	132	132	132	32
L-Cystine (g/kg)	3	3	3	3
Soybean Oil (g/kg)	70	70	70	17
Cellulose (g/kg)	50	50	50	50
Choline Bitartrate (g/kg)	2.5	2.5	2.5	2.5
Vitamin Mix (g/kg)	10	10	10	10
Mineral mix (g/kg)	35	35	35	35
t-Butylhydroquinone (g/kg)	0.014	0.014	0.014	0.014
Total energy (kcal)	4,000	4,000	4,000	3,800
Total protein (g/kg)	200	180	360	540
Total lipid (g/kg)	70.0	97.5	125.0	99.5
Energy (kcal/g)	4.0	4.0	4.0	3.8

*LD, low-dose red meat group; MD, medium-dose red meat group; HD, high-dose red meat group*.

Shortly after weaning and 1 week of adaption, the mice were randomly divided into four groups (half male and half female in each group) according to the red meat-based diet. The normal control group (*n* = 20) was given a standard diet (AIN-93G diet, without red meat, Table 1). A low-dose red meat group (*n* = 20) was given a diet with 25% red meat (Table 1), a medium-dose red meat group (*n* = 20) was given a diet with 50% red meat (Table 1), a high-dose red meat group (n = 20) was given a diet with 75% red meat (Table 1). All mice were fed *ad libitum*. Body weight was recorded weekly for each mouse. After 8 weeks, 10 mice per group were randomly anesthetized and sacrificed and colonic contents were immediately stored at −80°C for 16S rRNA gene sequencing.

The remaining mice in each group (*n* = 10) received drinking water containing 3% DSS for 9 days. The drinking bottles were checked every 2 days and the DSS solution was reconfigured to ensure that the concentration of DSS was the same over time. During the 9-day feeding, all the mice were continually fed the uniform standard diets (AIN-93G diet, without red meat) *ad libitum*. Body weight, food intakes, the presence of occult blood or gross blood in the feces, and stool consistency were recorded daily for each mouse during DSS (M.W 20000, Shanghai Aladdin Biochemical Technology Co., Ltd. China) induced colitis model. Mice of all DSS groups behaved in the same way. The disease activity index (DAI) was determined as previously described (14). Occult blood in feces was evaluated with Urine Fecal Occult Blood Test Kit (Yeasen Biotech Co., Ltd. China). On the 9th day, all of the mice were sacrificed, and whole colons were collected and measured length by a ruler. 16S rRNA gene sequencing

Total bacteria genome DNA from colonic contents was extracted with a Qiagen DNA Mini Kit (Qiagen, USA) and 16S rRNA genes were amplified used the specific primers of V3-V4: 341 Forward Primer: 5′-ACTCCTACGGGAGGCAGCAG-3′, 806 Reverse Primer: 5′-GGACTACHVGGGTWTCTAAT-3′. To remove contamination of samples with exogenous DNA or PCR products, PCR water instead of sample/template was used as negative controls. All PCR reactions were carried out in 30 μL reactions with 15 μL of Phusion High-Fidelity PCR Master Mix (New England Biolabs, USA), 0.2 μM of forward and reverse primers, and about 10 ng template DNA. Thermal cycling consisted of initial denaturation at 98°C for 1 min, followed by 30 cycles of denaturation at 98°C for 10 s, annealing at 50°C for 30 s, and extension at 72°C for 60 s, and a final extension at 72°C for 5 min. The PCR products were subjected to electrophoresis on 2% agarose gel. Samples with bright main strip between 400 and 450bp were chosen for further experiments. Then, the PCR products were purified with Gene JET Gel Extraction Kit (Thermo Scientific, USA). Sequencing libraries were generated using NEB Next®Ultra™DNA Library Prep Kit for Illumina (New England Biolabs, USA) following manufacturer's recommendations and index codes were added. The library quality was assessed on the Qubit@2.0 Fluorometer (Thermo Scientific, USA) and Agilent Bioanalyzer 2100 system. At last, the library was sequenced on an Illumina MiSeq platform and 250bp/300bp paired-end reads were generated. The data presented in the study are deposited in the NCBI SRA repository, accession number SRP310208.

To confirm differences in the abundances of individual taxonomy between groups, Metastats software was utilized. LEfSe was used for the quantitative analysis of biomarkers within different groups. To identify differences of microbial communities between groups, ANOSIM and MRPP (multi-response permutation procedure) were performed based on the Bray-Curtis dissimilarity distance matrices.

### Hematoxylin and Eosin (HE) Staining

The colon tissues were fixed in 4% paraformaldehyde, embedded in paraffin, and sectioned with the thickness of 5 μm. Then sections were stained with HE according to routine procedure. The histological score was evaluated by two independent investigators in a blinded fashion as previously described (15).

### Immunohistochemistry

Immunohistochemistry evaluation of the colon was performed as previously described (16). Briefly, the tissue sections were incubated with a primary antibody against inducible claudin1 (1:250, Abcam, USA) and evaluated after the incubation with a biotin-conjugated secondary antibody (1:800, ZSJQB Co., Ltd. Beijing).

### Real-Time Quantitative PCR (RT-PCR)

Total RNA was extracted from mice colon tissues using Trizol reagent (Takara, Japan). The cDNA was synthesized using a Revert Aid First Strand cDNA Synthesis kit (Thermo Scientific, USA). The mRNA levels of COX2, iNOS, NF-kB, ZO-1, claudin, and occludin were detected. The sequences of all primers are listed in Table 2. Quantitative real time PCR (RT-qPCR) was carried out on a CFX96 real-time PCR system (Bio-Rad, USA) by TB Green (Takara Bio, Japan). The two-step PCR amplification standard procedure was used: 95°C 30 s and 40 cycles of 95°C 5 s, 60°C 30 s and 72°C 30 s. To remove contamination of samples with exogenous DNA or PCR products, PCR water instead of sample/template was used as negative controls. Relative expression was calculated with 2^−ΔΔCt^ method using β-actin for normalization.

**Table 2:** PCR primers used in this study.

**Gene**	**Sense Primer (5^**′**^->3^**′**^)**	**Anti-sense Primer (3^**′**^->5^**′**^)**
β-actin	ATCACTATTGGCAACGAGCG	TCAGCAATGCCTGGGT ACAT
iNOS	GGAGCGAGTTGTGGATTG	CCAGGAAGTAGGTGAGGG
COX-2	GGCCTCGTGAGCTTCTTC	CTTCTGCAGTCCAGGTTCAA
NF-kB	ACCTTTGCTGGAAACACACC	ATGGCCTCGGAAGTTTCTTT
Claudin	CGACTCCTTGCTGAATCTGA	CGTGGTGTTGGGTAAGAGGT
Occludin	TGGCGGATATACAGACCCAA	CGATCGTGGCAATAAACACC
ZO-1	GAGTGGACTATCAAGTGAGCCTAA	ATCCAAGTTGCTCGTCAATCTAA

### Western Blotting

The colonic tissue was lysed in RIPA buffer (Thermo Scientific, USA) containing a protease inhibitor (Thermo Scientific, USA). Protein concentrations were measured with BCA protein assay kit (Thermo Scientific, USA). Equal amounts of protein samples were loaded to SDS-PAGE, and proteins were transferred to polyvinylidene fluoride membranes (Immobilon, USA). Following blocking with 5% non-fat milk for 1.5 h at room temperature, membranes were incubated with primary antibodies of IL-1β, TNF-α, IL-17, IL-6, NF-kB, and β-actin (Abcam, USA) overnight at 4°C. Then the membranes were incubated with HRP-conjugated secondary antibody (1:1000, ZSJQB Co., Ltd. Beijing) for 2 h at room temperature. Finally, enhanced chemiluminescence detection kit (Thermo Scientific, USA) was used for color development. The bands were detected with a Bio-Rad Western blot detection system (Bio-Rad ChemiDoc XRS+ imager, Bio-Rad Laboratories, USA), and β-actin was used as the internal control.

### Statistical Analysis

All statistical analysis was performed using IBM SPSS software (IBM SPSS Statistics for Windows, version 21.0). Measurement data were presented as mean ± standard deviation (SD). Differences between groups were evaluated using univariate analysis of variance (ANOVA) and least significant difference *post-hoc* tests. Each experiment was repeated three times. A *p*-value < 0.05 was considered statistically significant.

## Results

### High Red Meat Intake Alters the Composition of Gut Microbiota

**Figure 1:**
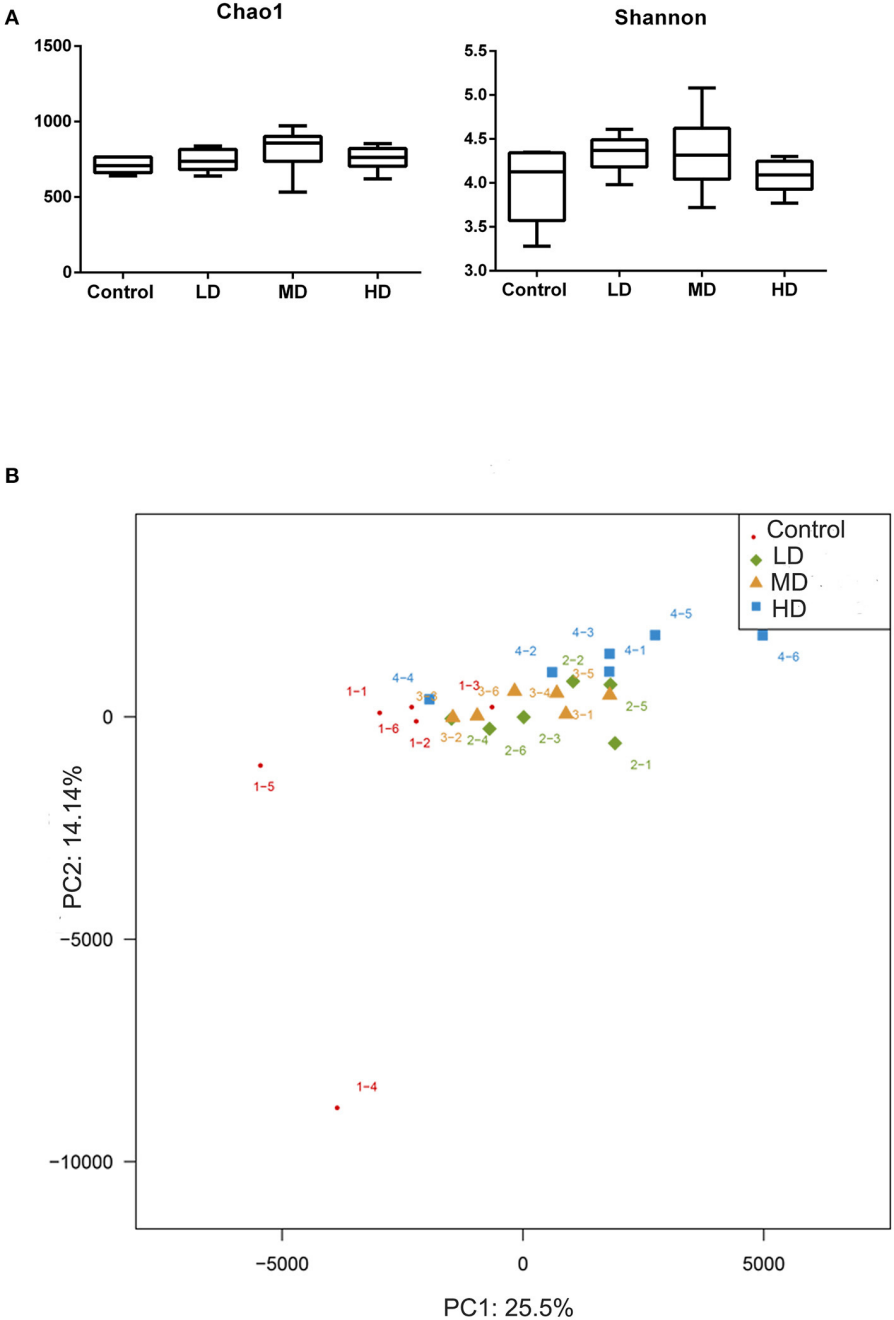
Correlation of high red meat intake with gut microbiota. **(A)** The abundance and diversity of microbiota (Chao 1 and shannon index) in mice fed with different levels of red meat diets. **(B)** Principal Component Analysis (PCA) of microbiota in mice fed with different levels of red meat diets. LD, low-dose red meat group; MD, medium-dose red meat group; HD, high-dose red meat group.

**Figure 2:**
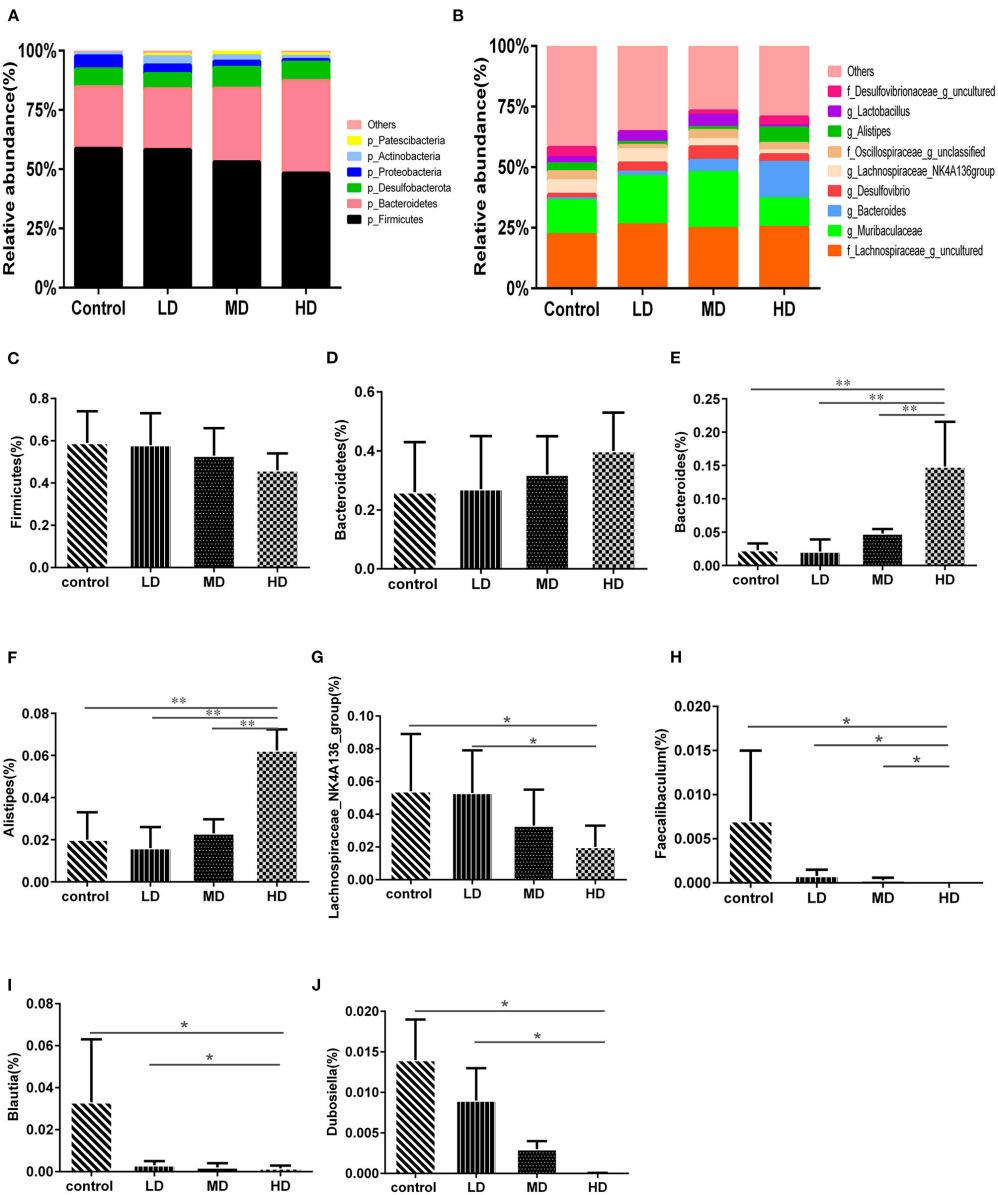
High red meat intake alters gut microbiota. **(A)** The phylum level of microbiota composition and comparison of microbiota in mice fed with different levels of red meat diets: **(C)** *Firmicutes*, **(D)** *Bacteroidetes*. **(B)** The genus level of composition and comparison of microbiota in mice fed with different levels of red meat diets: **(E)** *Bacteroides*, **(F)** *Alistipes*, **(G)** *Lachnospiraceae_NK4A136_group*, **(H)** *Faecalibaculum*, **(I)** *Blautia*, **(J)** *Dubosiella*. The chart data are presented as mean ± SD, and *p < 0.05, **p < 0.01. LD, low-dose red meat group; MD, medium-dose red meat group; HD, high-dose red meat group.

**Figure 3:**
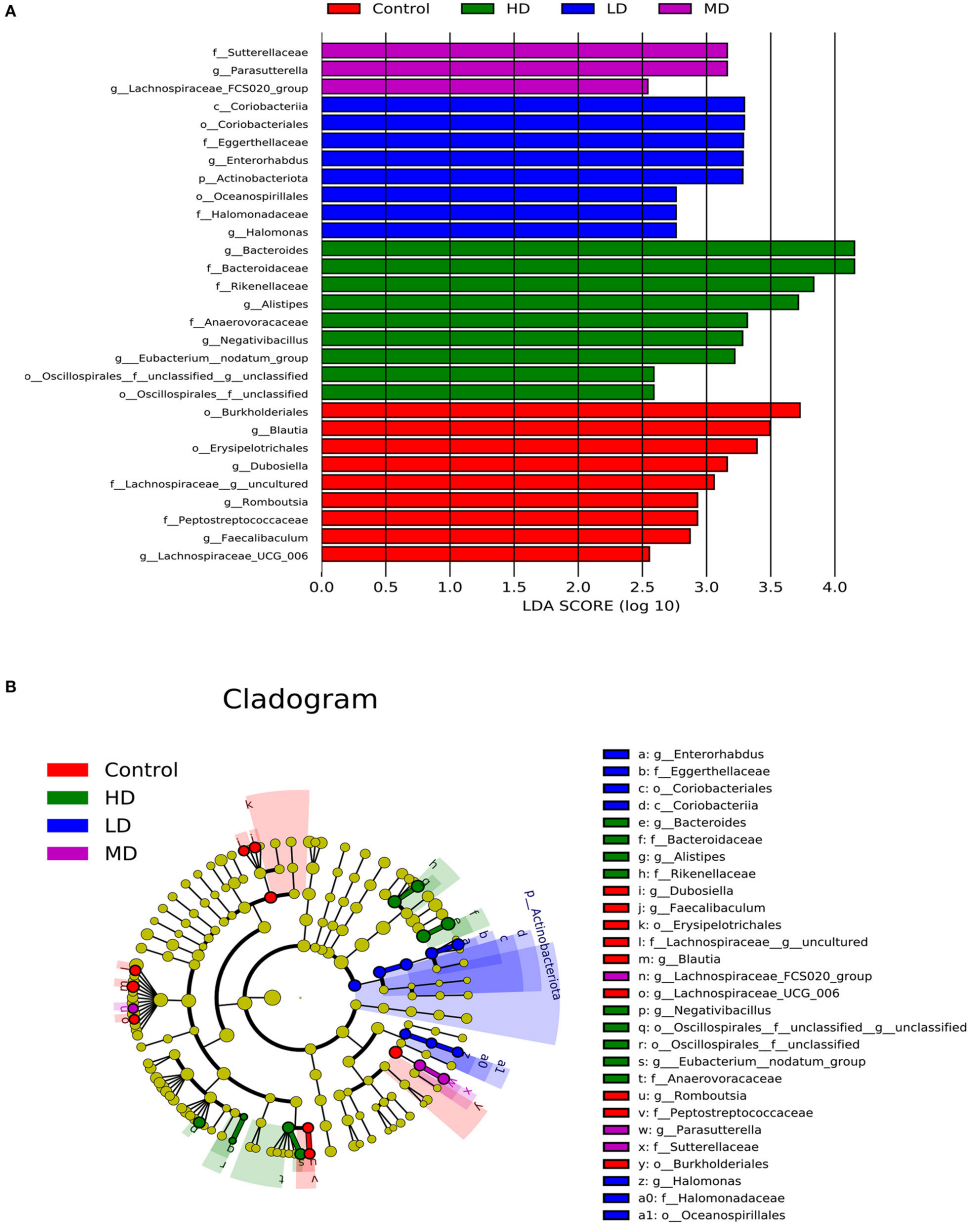
Characteristics of microbial community composition using LEfSe (LDA Effect Size) analysis. **(A)** The differential bacteria of the gut microbiota meeting a significant LDA threshold value of >2.5 between groups with different levels of red meat diet. **(B)** The cladogram based on LEfSe shows differential bacteria of the gut microbiota between groups with different levels of red meat diet.

Mice were fed with different levels of red meat diets for 8 weeks and the colonic contents were analyzed by 16S rRNA gene sequencing. The measured values of the abundance of microbiota (Chao1) and the microbiota diversity (Shannon) were shown in Figure 1A. We found that the microbiota abundance and diversity did not change significantly. Principal Component Analysis (PCA), which reflects the difference in gut microbiota between groups based on different levels of red meat diet (Figure 1B), showed that red meat diet altered the gut microbiota. Microbial community barplot showed that the microbiota composition of phylum and genus changed significantly (Figures 2A,B). At the phylum level, the major phyla of gut microbiota were *Firmicutes, Bacteroidetes, Desulfobacterota, Proteobacteria*, and *Actinobacteria*. Meanwhile, we found the relative abundance of phylum *Firmicutes* decreased gradually and the phylum *Bacteroidetes* increased gradually in the high-dose red meat groups compared to the controls or low-dose red meat group, but there was no statistically significant difference (Figures 2C,D). At the genus level, the relative abundance of genera *Bacteroides* and *Alistipes* increased significantly in the high-dose red meat groups compared to the controls and low-dose red meat groups (Figures 2E,F, *P* < 0.01), and the genera of *Lachnospiraceae_NK4A136_group* decreased significantly in the high-dose red meat groups compared to the controls (Figure 2G, *P* < 0.05). Meanwhile, we found that the genera of *Faecalibaculum, Blautia* and *Dubosiella* decreased significantly in the high-dose red meat groups compared to the controls (Figures 2H–J, *P* < 0.05). The changes of gut microbiota within different levels of red meat diets groups were further analyzed by LEfSe. In our study, a total of 29 differentially abundant taxons (from phylum to genus level) were found between the four groups (Figure 3).

### High Red Meat Intake Leads to Malnutrition and Enhances DSS-Induced Colitis

**Figure 4:**
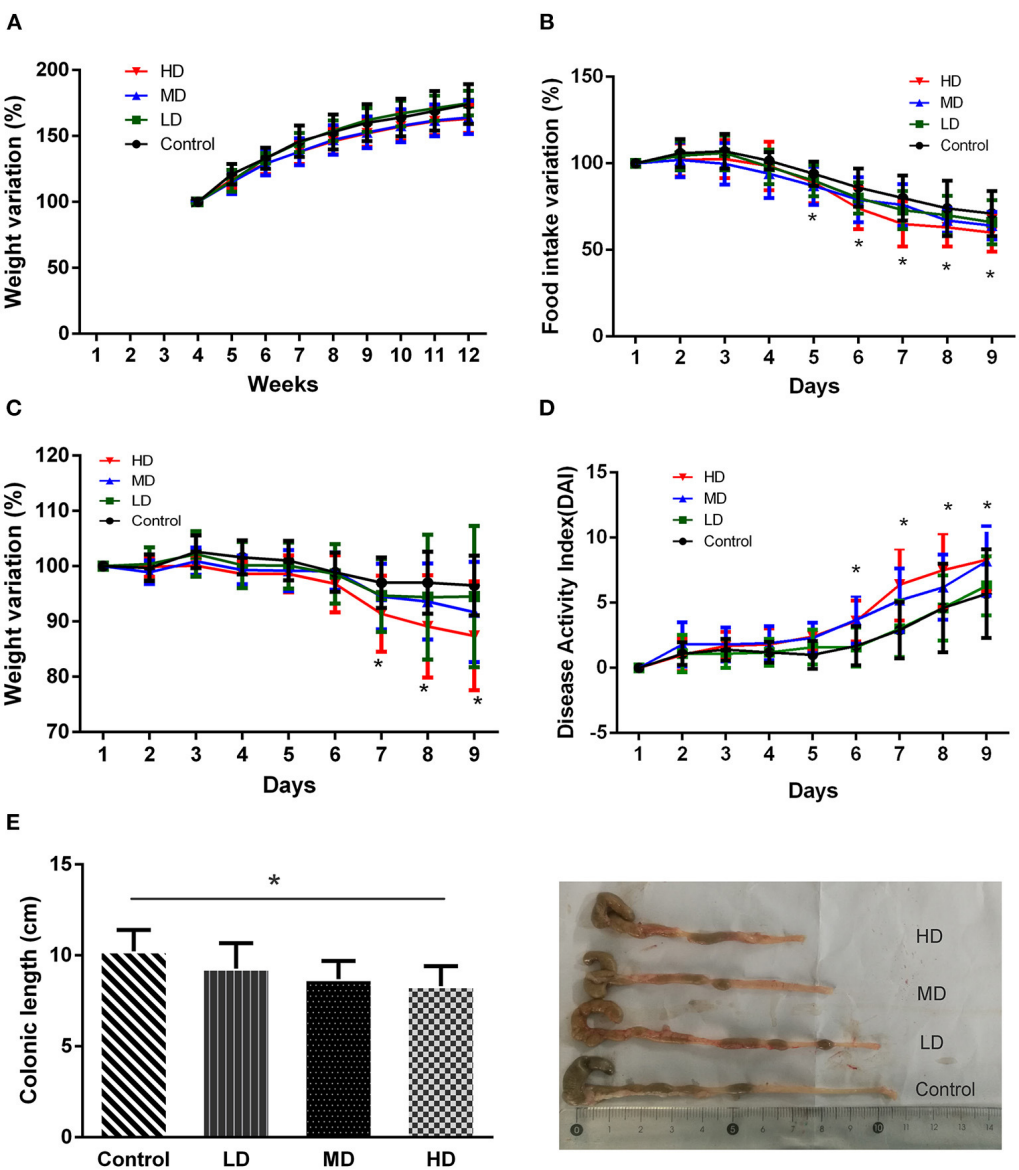
Effects of different levels of red meat diets. **(A)** Body weight variation during 8 weeks of feeding with different levels of red meat diets. **(B)** Food intake variation of different levels of red meat diets on DSS colitis. **(C)** Body weight variation of different levels of red meat diets on DSS colitis. **(D)** Disease activity index of different levels of red meat diets on DSS colitis. **(E)** Comparison of different red meat diets on colonic length and representative images of DSS colitis mice. Data are presented as mean ± SD, and *indicates *p* < 0.05.

**Figure 5:**
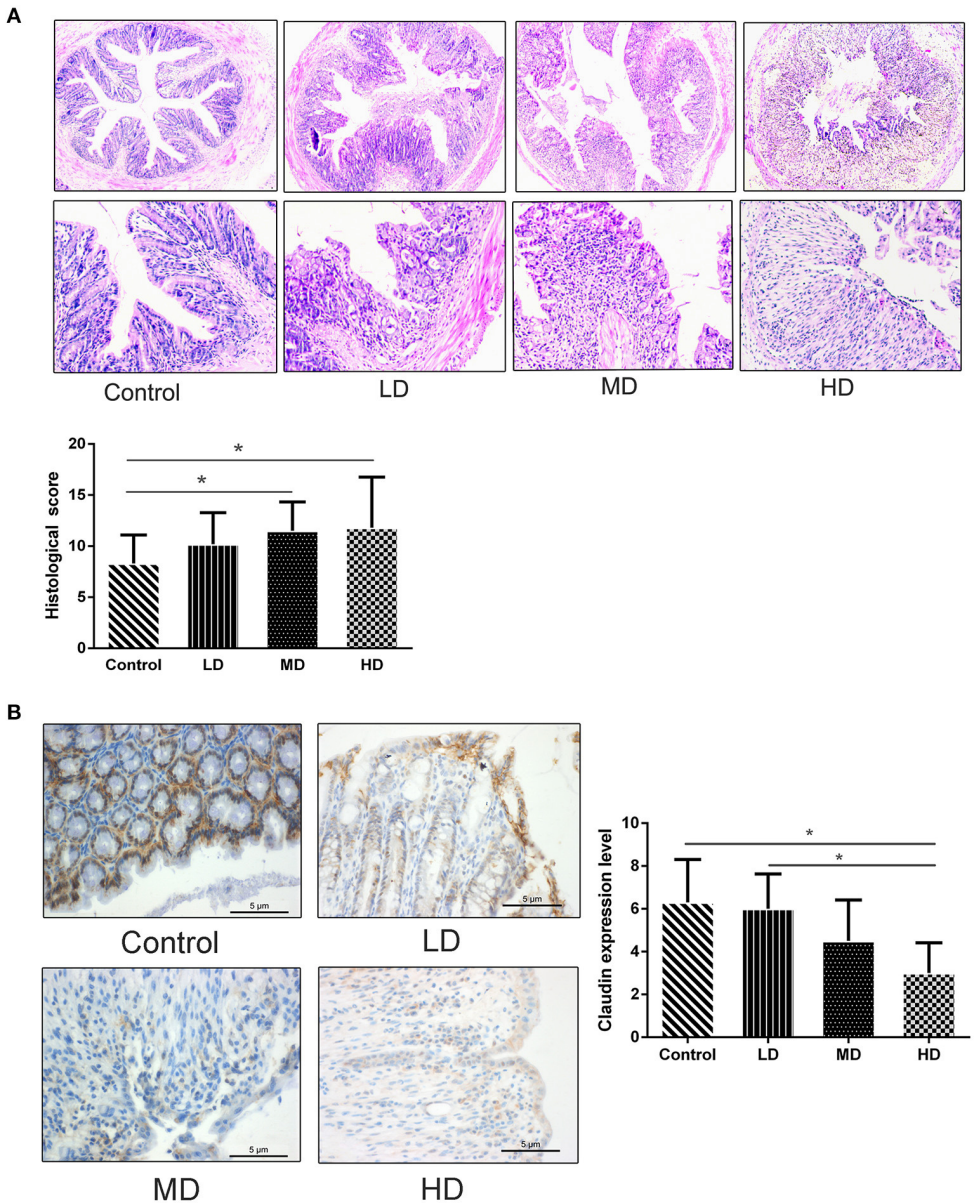
HE staining and immunohistochemical analysis in the colon tissue of DSS colitis. **(A)** Representative images of HE staining of the colon tissues and histologic injury scored for HE staining in mice fed with various levels of red meat diets. Magnifications: × 100 and × 200. **(B)** Immunohistochemical analysis of Claudin-1 expression in the colon tissue of mice fed with various levels of red meat diets. Magnifications: × 400. Data are presented as mean ± SD, and *p < 0.05.

During the 8 weeks of feeding with red meat diets, the body weights of mice decreased a little compared to the controls, but there was no statistical significance (Figure 4A). To evaluate effects of dietary red meat on colonic inflammation, we exposed mice fed with different levels of red meat-based diets to DSS (17), which is a widely used to induce colitis resembling IBD. The food intake variation of mice after 3.0% DSS solution of drinking water in different groups is shown in Figure 4B. When the mice were exposed to DSS in early3 days, the food intake changed slightly. However, during the last 5 days, the food intake reduced significantly in high-dose red meat group compared with the control group (*p* < 0.05). The body weight variation of mice after 3.0% DSS solution of drinking water is shown in Figure 4C. During the first 5 days, the body weight changed slightly, but during the last 3 days, the body weight reduced significantly in high-dose red meat group compared with the control group (*p* < 0.05). Significant reductions in food intake and body weight seem to indicate malnutrition in mice. DAI score is a comprehensive score to assess the severity of colitis. We found at the last 4 days of the experiment, the DAI score of high-dose red meat group increased significantly compared with the control group after 3.0% DSS induced colitis (*p* < 0.05, Figure 4D). Our results also showed that high-dose red meat group significantly shortened the colonic length compared with the control group after 3.0% DSS induced colitis (*p* < 0.05, Figure 4E). By HE staining, we observed that the high and medium-dose red meat groups exhibited more severe epithelial damage and higher levels of inflammation infiltration compared with the control groups (Figure 5A). In conclusion, our data demonstrates that high intake of red meat diet enhances colonic inflammation of DSS-induced colitis.

### High Red Meat Intake Impairs the Colon Barrier Integrity in DSS-Induced Colitis

**Figure 6:**
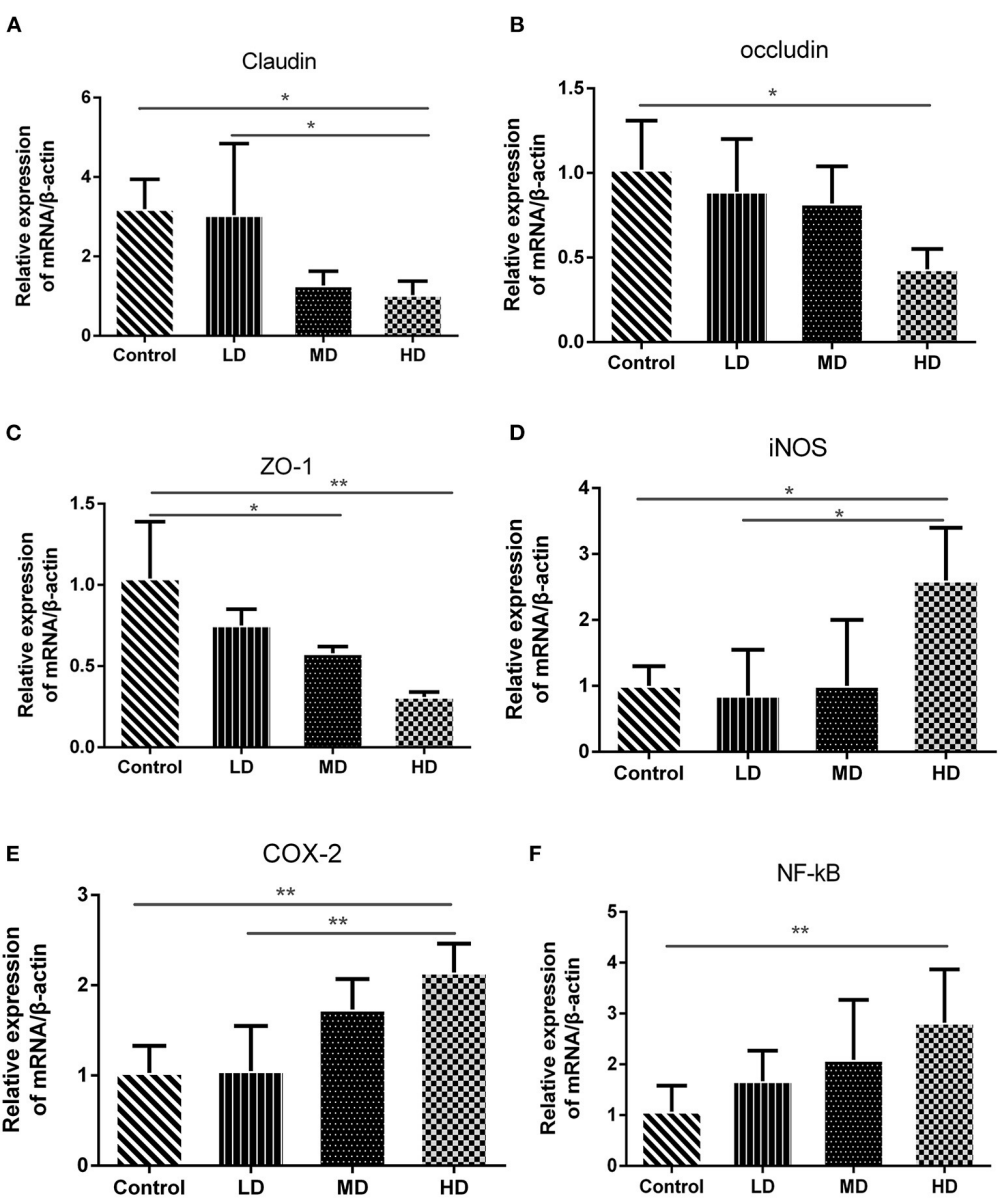
Gene expression levels determined by RT-PCR. The mRNA level of **(A)** Claudin, **(B)** Occludin, **(C)** ZO-1, **(D)** COX-2, **(E)** iNOS, and **(F)** NF-kB. Data are presented as mean ± SD, and *p < 0.05, **p < 0.01.

The epithelial cells and tight junctions contribute to intestinal barrier integrity in conjunction with the mucus secreted from the goblet cells and antimicrobial factors from the immune system cells (18). Tight junctions are critical in preventing the entry of the microbial toxins, antigens and other harmful substances (19). Immunohistochemistry evaluation showed that the expression of tight junction proteins-claudin1 was significantly reduced in high-dose red meat groups compared with the control and low-dose red meat groups (Figure 5B). Meanwhile, we found the relative mRNA expression of *claudin, occludin*, and *ZO-1* was also decreased in high-dose red meat groups compared with the control or low-dose red meat groups (Figures 6A–C). These experiments indicate that high-dose red meat diet could impair colonic barrier integrity in DSS-induced colitis.

### High Red Meat Intake Exacerbates Colonic Inflammation in DSS-Induced Colitis

**Figure 7:**
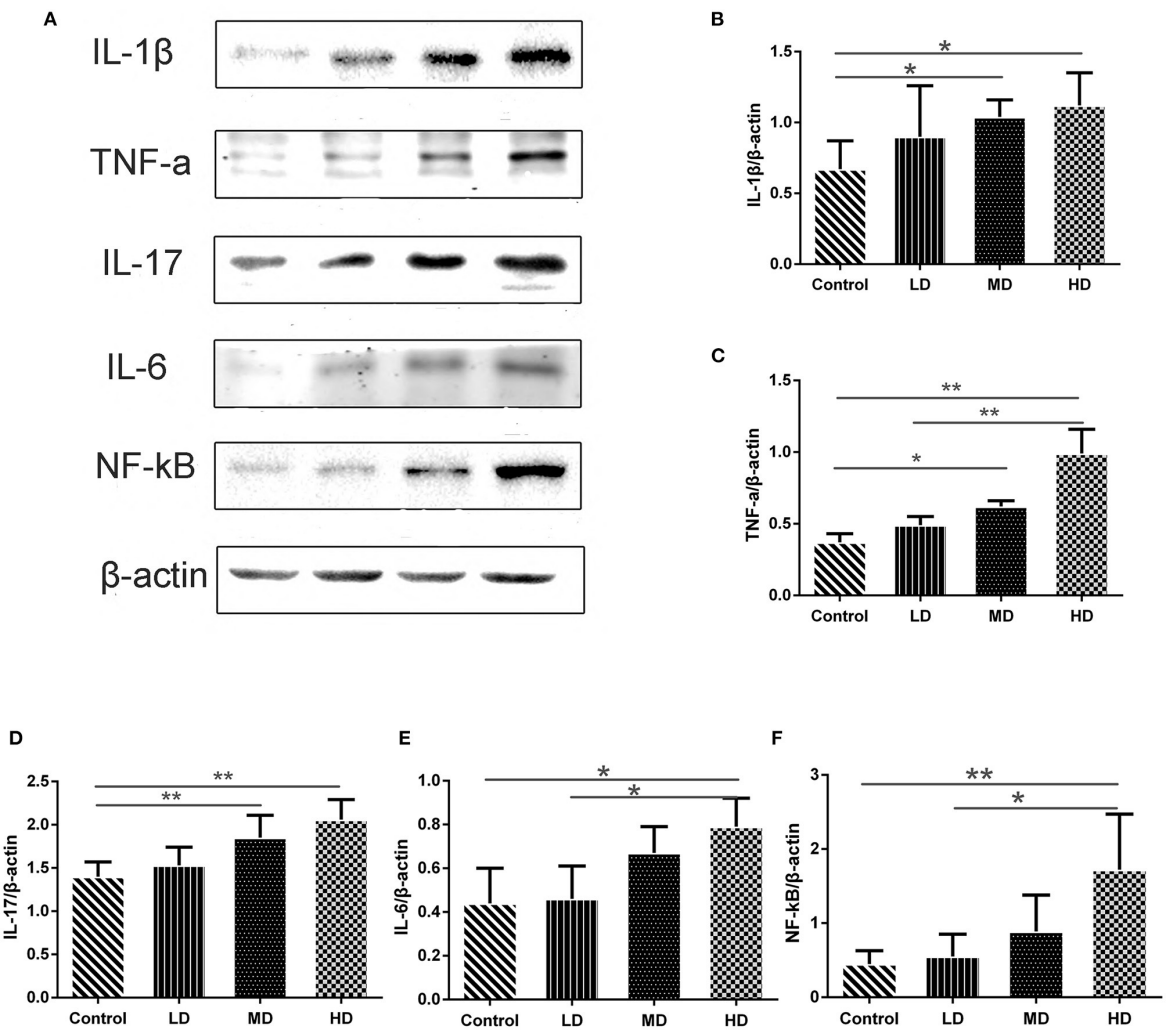
Gene expression levels determined by Western blot. **(A)** Representative pictures of Western blot bands and Western blot analysis showed the protein expression comparison of **(B)** IL-1β, **(C)** TNF-α, **(D)** IL-17, **(E)** IL-6, and **(F)** NF-kB, and β-actin was used as the internal control. Data are presented as mean ± SD, and *p < 0.05, **p < 0.01.

High meat protein diet induces colonic inflammation and upregulates several key cytokines including IL-1β, TNF-α, IL-6 (20). High fat diet impairs the intestinal immune system and increases sensitivity to DSS (21). High meat heme reduces fecal butyrate and further exacerbates DSS-induced colitis (13). To determine whether high red meat intake exacerbates colonic inflammation in DSS-induced colitis, RT-PCR and Western blot were performed. We observed that relative mRNA expression of iNOS and COX-2 was significantly higher in the high-dose red meat group than in the control group or the low-dose red meat group (Figures 6D,E). We also noticed that NF-kB p65 mRNA was expressed at higher levels in the high-dose red meat groups than control group (Figure 6F). Besides, pro-inflammatory cytokines including IL-1β, TNF-α, IL-17, and IL-6 were significantly increased in high-dose red meat groups compared with the control group (Figures 7A–E). Moreover, western blotting also showed an increase of NF-kB p65 protein in the high and medium-dose red meat groups than control group (Figures 7A,F). All of above results suggest that high-dose red meat diet significantly increases colonic inflammation in the DSS-induced colitis mouse model.

## Discussion

Epidemiological study has shown that the prevalence and incidence of IBD is highest in Western countries (22). In particular, the prevalence and incidence of IBD in immigrants of Western countries increases significantly, far exceeding those in their country of origin (23). More evidence suggests that Western diets, such as high red meat intake, are associated with increased incidence of IBD (7). Eating habits can affect microbiota in the human gut, and gut microbiota can respond to altered diets (24). Different dietary components, such as protein (25), fat, fiber (26), salt, amino acids, heme, etc., can alter the intestinal microbiota, thereby affecting intestinal permeability and inflammation. However, how the red meat diet affects the gut microbiota is still not clear.

In this study, we found that the mostly increased nutrient in high red meat diets was protein. The total protein content of MD (36%) and HD (54%) compared to the control group (20%) almost increased exponentially. It has been shown that long-term high-protein diets can reduce body weight, alert metabolism and intestinal function (27). Most excessive dietary protein can lead to significant weight loss in mice with colitis, especially casein, isolated soy protein and whey (26). Similarly, our study also found that long-term high red meat diets led to reduced intake and body weight significantly during DSS-induced colitis, which further led to malnutrition. Malnutrition is highly prevalent in IBD patients and the reported prevalence of malnutrition in IBD patients ranges between 20 and 85% (28). Malnutrition often indicates more severe colitis and poor clinical outcomes (29).

High-protein diets can also affect the intestinal microbiota. In this study, we analyzed the gut microbiota in mice fed with different levels of red meats by 16S rRNA gene sequencing. We found that there was no difference in bacterial alpha diversity between the four groups, but the composition of microbiota changed significantly. This is consistent with the results of Klara Kostovcikova et al., which found that a protein-rich diet led to gut microbiota disorders and promoted colitis in mice (30).

The importance of the intestinal microbiota in the early life has been established. During childhood, different dietary habits can alert intestinal microbiota, and these intestinal microbiota stabilizes and becomes a balanced system in adulthood (31, 32). The intestinal microbiota of mice fed with different levels of red meat in early developmental stage changes and becomes stable. Therefore, we provided a uniform diet during the DSS-induced colitis.

We found that at the phylum level, high red meat intake reduced the relative abundance of *Firmicutes*, and increased the abundance of *Bacteroidetes*. Our findings about phylum level changes were consistent with Matijašić's study (33), which revealed that the abundance of *Firmicutes* significantly decreased and *Bacteroidetes* significantly increased in patients with UC and CD. In another experimental study on diet and gut microbiota (30), it was also found that high intake of animal protein diet reduced the relative abundance of *Firmicutes*, which was in agreement with our findings.

In this study, we found at the genus level, high red meat intake significantly increased the relative abundance of *Bacteroides* and *Alistipes* genera in colon, but reduced the abundance of *Lachnospiraceae_NK4A136 group, Faecalibaculum, Blautia*, and *Dubosiella* genera. Previous studies have shown that the genera of *Bacteroides* and *Alistipes* can affect amino acid transport, metabolism, energy production and transport, and inflammation (34–36). *Bacteroides* can secret metalloprotease toxin both *in vivo* and *in vitro* (37), which stimulate the cleavage of intercellular adhesion protein E-cadherin in colonic epithelial cells, resulting in increased colonic mucosal permeability and activating NF-kB pathway. The genus *Alistipes* is considered as a kind of pathogenic bacteria. It has been shown that the genus *Alistipes* was correlated with obesity (38), diabetes (39), constipation, colitis and colorectal cancer (40). Schirmer et al. (41) found that the relative abundance of *Bacteroides* and *Alistipes* in IBD patients was increased.

*Lachnospiraceae_NK4A136 group* is butyrate-producing bacteria (42), and butyrate is a beneficial substance in the gut, which protects the intestinal mucosa, regulates immunity, and reduces inflammation. So, the reduction of *Lachnospiraceae_NK4A136– group* will be detrimental to the integrity of the intestinal mucosa and can easily lead to intestinal inflammation. Some studies have found that the genus *Lachnospiraceae_NK4A136 group* have effects on the prevention and treatment of depressive symptoms (43), acute colitis (44), type 2 diabetes and obesity. In our experiment, we found that high intake of red meat diet reduced the relative abundance of *Lachnospiraceae_NK4A136_group* compared with the control group.

Both *Faecalibaculum* and *Blautia* are considered to be potential probiotics, and both of them can produce short-chain fatty acids (SCFAs). *Faecalibaculum* can produce butyrate, which promotes the differentiation of Treg cells in the colon to reduce inflammation (45). It has been found that the relative abundance of *Faecalibaculum* in patients with IBD was increased (46). Some studies have also found the reduction of *Faecalibaculum* is related with the occurrence of colorectal cancer (47), allergies, asthma (48), and rheumatoid arthritis (49). In our study, we also found high intake of red meat diet reduced the relative abundance of *Faecalibaculum*.

The genus *Blautia* has been confirmed to kill harmful microorganisms in the intestines, thereby greatly preventing infections (50). Studies have found that the reduction of genus *Blautia* is associated with IBD (51), colorectal cancer (52), diabetes and obesity (53), and major depressive disorder (54). Our results showed that high intake of red meat diet reduced the relative abundance of *Blautia*. Meanwhile, high intake of red meat diet reduced the relative abundance of *Dubosiella*. Qiu et al. (55) showed that *Dubosiella* played a novel role in regulating SCFAs production and obesity.

Microbiota composition in the gut not only affects the production of the metabolites produced by the microbiota but also affects the immune system, and then aggravates the colonic inflammation (56). Study on colitis of animal models has suggested that colonic inflammation relies heavily on a triggering event mediated by the gut microbiota (57). Recent study has identified over 200 IBD risk loci in the IBD patients (58). It has been postulated that gut microbiota interacts with these risk loci, resulting in gut microbiota disorders and subsequently leading to the occurrence of IBD. The association of gut microbial disorders and the subsequent dysregulated immune response leads to ThI/Th2 immune response and the progression of IBD (58). In our study, we found that high intake of red meat diet led to gut microbiota disorders. Furthermore, we found that the sensitivity of mice with higher red meat intake to DSS-induced colitis was enhanced, resulting in faster body weight loss, shortened colonic length, severe histological colon damage and colon barrier integrity loss. Moreover, it also increased pro-inflammatory cytokines including IL-1β, TNF-α, IL-17, IL-6, and inflammatory inducible enzymes such as COX-2 and iNOS.

Apart from the gut microbiota, other crucial factors that are also involved in the maintenance of intestinal homeostasis include intestinal barrier integrity and permeability, because dysfunction of intestinal barrier integrity and permeability are common symptoms in patients with IBD (59). Tight junction proteins are critical for intestinal barrier integrity and can regulate paracellular permeability (60). The tight junction protein is mainly composed of claudin, occludin and ZO-1 (61). Studies have found that patients with UC have reduced expression of tight junction proteins (claudin, occludin and ZO-1) in the colonic epithelium (62), suggesting that tight junction proteins may be potential markers of colonic epithelial cell integrity. In our study, we also found that high intake of red meat diet decreased the expression of claudin, occludin and ZO-1 and upregulated of nuclear transcription factor NF-kB. The upregulation of NF-kB correlated significantly with the severity of intestinal inflammation.

In colonic inflammation, a number of pro-inflammatory cytokines are produced, such as IL-1β, TNF-α, IL-17, and IL-6 (63). The nuclear transcription factor NF-kB affects the pathogenesis of colitis and regulates the expression of some pro-inflammatory cytokines (64) and inducible enzymes such as COX-2 and iNOS (65). These results indicate that high intake of red meat diet exacerbates experimental colitis in mice. Our study is consistent with the recently published articles (25, 30), which demonstrates that increased animal protein diet promotes the susceptibility to colitis by gut microbiota disorders.

In summary, our results suggest that high red meat intake can induce the destruction of gut microbiota, which in turn impairs the colon barrier integrity and aggravates DSS-induced colitis. Our results may provide more insight into the relationship between dietary interventions with IBD and suggest that an optimized diet, reasonable nutrients, and appropriate red meat intake may prevent the occurrence of IBD.

## Data Availability Statement

The data presented in the study are deposited in the NCBI SRA repository, accession number SRP310208.

## Ethics Statement

The animal study was reviewed and approved by the Ethics Committee of Xinjiang Medical University (approval no. IACUC20180411-04).

## Author Contributions

D-pL: study Design, data collection, and manuscript preparation. MC: study design and data collection. FT: statistical analysis and data interpretation. X-yL: literature search. PY: study design and funds collection. All authors contributed to the article and approved the submitted version.

## Conflict of Interest

The authors declare that the research was conducted in the absence of any commercial or financial relationships that could be construed as a potential conflict of interest.
